# Cellular and Molecular Strategies in Cyanobacterial Survival—“In Memory of Prof. Dr. Wolfgang Lockau”

**DOI:** 10.3390/life11020132

**Published:** 2021-02-09

**Authors:** Khaled A. Selim, Iris Maldener

**Affiliations:** Organismic Interactions Department, Interfaculty Institute for Microbiology and Infection Medicine, Cluster of Excellence ‘Controlling Microbes to Fight Infections’, Tübingen University, Auf der Morgenstelle 28, 72076 Tübingen, Germany

Aerobic life on Earth evolved about 3.8–2.7 billion years ago with the evolution of oxygenic photosynthesis by cyanobacteria. Approximately 2.4 billion years ago, the ancestors of today’s cyanobacteria were the first oxygenic photoautotrophs to release molecular O_2_ as a waste product of the oxygenic photosynthesis. Via endosymbiosis, the photosynthetic ability was later transmitted from cyanobacteria to eukaryotes, giving rise to plastids of higher plants. Nowadays, cyanobacteria display diverse cell morphologies and occupy almost all illuminated aquatic and terrestrial habitats, including harsh environments like deserts, oceans, hypersaline, volcanic and thermal biospheres; therefore, they represent one of the quantitatively most abundant organisms on earth.

Cyanobacteria are the most influential photoautotrophic prokaryotes that can perform photosynthesis and fix atmospheric carbon dioxide via the Calvin–Benson cycle for primary metabolism. In addition, several species can fix atmospheric nitrogen (N_2_). As primary producers, cyanobacteria play a key role in the global carbon cycle. In addition, the diazotrophic cyanobacteria are the dominant nitrogen fixers in the oceans, introducing combined nitrogen into the global nitrogen cycle. The carbon/nitrogen assimilation reactions require a tight regulation and a constant sensing of the quantity and quality of the carbon and nitrogen availability. Generally, both metabolisms are coordinated by a complex crosstalk between different input signals. The sensing and regulation of the nitrogen/carbon metabolism in cyanobacteria depend on several signal-transduction proteins, which transduce the energy/carbon/nitrogen/day–night status of the cell and include several adaptation strategies. For example, chlorosis in case of nitrogen starvation in unicellular cyanobacteria or a carbon concentrating mechanism to cope with low levels of atmospheric CO_2_ and the slow rate of the CO_2_ fixing enzyme. Another strategy to cope with nitrogen starvation is realized by filamentous species, which show a division of labor between different cell types, the photosynthetic active vegetative cells and the N_2_ fixing heterocysts. These bacteria represent true multicellular organisms which are able to communicate via special cell–cell connections: the septal junctions.

Some of the filamentous heterocyst-forming cyanobacteria differentiate another cell type, the resistant spore-like akinetes, which allow survival under instances of cold and nutrient starvation. When environmental conditions become favorable, these akinetes can germinate and regrow to long filaments. To find and occupy the optimal environment, cyanobacteria deploy yet another strategy: they form small filamentous hormogonia that display pili-based motility. This third differentiated motile cell type is also involved in establishing the plant–cyanobacterium symbiosis.

In this Special Issue of *Life* on cyanobacterial cellular and molecular strategies for survival, we summarize some of the recent research advances in cyanobacterial biology in a wonderful collection of original research and review articles. By mutational studies, Khudyakov et al. [1] investigated the influence of three small proteins (PatS, PatX and HetN) on HetR, the master regulator of heterocyst differentiation in the multicellular cyanobacterium *Nostoc* sp. PCC 7120. PatS, PatX and HetN are negative regulators of HetR with a conserved pentapeptide motif RG(S/T)GR. The paper demonstrates the importance of a tight control of HetR activity for vegetative growth and heterocyst pattern formation. Zulkefli and Hwang [2] investigated the effect of various inorganic nitrogen sources on the growth of *Anabaena variabilis* under nitrogen fixing conditions and on heterocyst differentiation. Álvarez-Escribano et al. [3] analyzed a new example of post-transcriptional regulation by a small regulatory RNA, NsrR1 (nitrogen stress-repressed RNA 1), and identified the gene *all1871*, which is required for the diazotrophic growth of *Nostoc* sp. PCC 7120. The heterocyst specific expression of *all1871* is regulated by direct binding of NsrR1 to the 5′ untranslated region of the *all1871* mRNA and represents an example of indirect regulation via the global nitrogen regulator NtcA.

Regarding photosynthesis and carbon metabolism, Gollan et al. [4] described the global transcriptional changes in the *Nostoc* sp. PCC 7120 upon the shift from high carbon (3% CO_2_) to low carbon (0.04% CO_2_) conditions. Selim and Haffner [5] studied the effects of long-term exposure of heavy metal stress on the nitrogen-starved unicellular cyanobacterium *Synechococcus elongatus* PCC 7942. Thurotte et al. [6] showed that the DnaK3 chaperon is required for efficient photosynthesis and for the biogenesis of thylakoid membranes, where the photosynthetic machinery is localized in the unicellular cyanobacterium *Synechocystis* sp. PCC 6803. Margulis et al. [7] demonstrated that overexpression of the gene encoding for Pgr5-like protein, which is required for cyclic photosynthetic electron flow paths in eukaryotes, causes accumulation of chlorophyll, photosystems, and glycogen contents in *Synechocystis* sp. PCC 6803 and is involved in redox regulation. Sun et al. [8] explored the influence of the circadian clock on the dynamic regulation of CO_2_ fixation and the carboxysome (CO_2_-fixing microcompartment) localization in response to diurnal day–night rhythm in *Synechococcus elongatus* PCC 7942. Koch et al. [9] studied the role of the reserve carbon biopolymer polyhydroxybutyrate (PHB) in *Synechocystis* sp. PCC 6803 physiology. They showed that the intracellular PHB content increases under diurnal day–night cycles.

Besides these original research papers, we received several comprehensive reviews summarizing the updates of some important aspects of cyanobacterial biology. Springstein et al. [10] reviewed the structural determinants and their role in cyanobacterial morphogenesis. Brandenburg and Klähn [11] described the role of small regulatory proteins on cyanobacterial metabolism, while Labella et al. [12] focused on the signaling roles of another small regulatory protein PipX in the coordination of nitrogen metabolism. Rachedi et al. [13] provided a mechanistic overview of stress signaling in cyanobacteria. Conradi et al. [14] and Schirmacher et al. [15] summarized the current advances on the role of pili in motility and natural competence in cyanobacteria. Finally, Lima et al. [16] described the cyanobacterial secretion systems with special focus on extracellular vesicles.

We expect that this collection will provide the reader with a comprehensive overview of some important aspects of cyanobacterial biology. We are confident that this cyanobacterial Special Issue of *Life* will attract the attention of a broad audience, in particular, of any scientist interested in fundamental biological questions, including aspects of cyanobacterial biology.

We would like to dedicate this Special Issue of *Life* to Prof. Dr. Wolfgang Lockau, for his distinguished contribution to the biochemistry and cell biology of cyanobacteria.
